# Integrated Metabolome and Transcriptome Analysis Reveals a Regulatory Network of Fruit Peel Pigmentation in Eggplant (*Solanum melongena* L.)

**DOI:** 10.3390/ijms232113475

**Published:** 2022-11-03

**Authors:** Xiaohui Zhou, Songyu Liu, Yan Yang, Jun Liu, Yong Zhuang

**Affiliations:** Institute of Vegetable Crops, Jiangsu Academy of Agricultural Sciences, Jiangsu Key Laboratory for Horticultural Crop Genetic Improvement, Nanjing 210014, China

**Keywords:** eggplant, fruit peel color, flavonoids, anthocyanin, metabolome, transcriptome

## Abstract

The color of fruit peel is an economically important character of eggplant, and black-purple eggplant has received much attention for being rich in anthocyanin. However, the reason why different fruit peel colors form in eggplant is not well understood. In the present study, an integrative analysis of the metabolome and transcriptome profiles was performed in five eggplant varieties with different fruit colors. A total of 260 flavonoids were identified, and most of them showed significantly higher abundance in black-purple varieties than in other varieties. The transcriptome analysis indicated the activation of early phenylpropanoid biosynthesis genes (*SmPAL*, *SmC4H,* and *Sm4CL*) was more responsible for anthocyanin accumulation, while *SmF3′5′H* was the key factor for the formation of a purple color. Furthermore, two transcription factors, *SmGL2* and *SmGATA26,* were identified as new hub genes associated with anthocyanin accumulation. The silencing of *SmGL2* and *SmGATA26* reduced anthocyanin accumulation in eggplant fruit peels, suggesting the possible involvement of *SmGL2* and *SmGATA26* in regulating anthocyanin biosynthesis. In addition, the pathway of plant hormone signal transduction was significantly enriched, indicating that phytohormones may cooperatively interact to modulate flavonoid biosynthesis. This study provides comprehensive information of flavonoid metabolites and new insights into the regulatory network of fruit coloration, which might be useful for the molecular breeding of eggplant.

## 1. Introduction

Flavonoids, as important secondary metabolites, are widely distributed in plants. They are involved in many biological functions and play crucial roles in plant pigmentation and protection against biotic and abiotic stress [1]. Flavonoids mainly contain seven groups, including flavonols, flavones, flavanones, flavanols, isoflavones, chalcones, and anthocyanins. Meanwhile, various modifications (glycosylation, acylation, and others) and molecular polymerization lead to the formation of a great number of flavonoid compounds [2]. Anthocyanins are the most important water-soluble pigments in horticultural plants and confer a red-to-blue coloration to plant tissues [3]. Moreover, the copigmentation initiated by flavones and flavonols as well as glycosides increases the stability of the colored structural forms of anthocyanins and consequently enhances the final coloration [4,5].

Flavonoids/anthocyanins are synthesized via the phenylpropanoid and flavonoid pathways, which involve at least two types of genes: structural genes and regulatory genes [2]. Structural genes encode the enzymes that directly participate in flavonoid biosynthesis, including phenylalanine ammonia lyase (*PAL*), cinnamate 4-hydroxylase (*C4H*), 4-coumarate-CoA ligase (*4CL*), chalcone synthase (*CHS*), chalcone isomerase (*CHI*), flavanone 3-hydroxyl enzyme (*F3H*), dihydroflavonol reductase (*DFR*), anthocyanin synthase (*ANS*), and flavonoid 3-O-glucosyltransferase (*UFGT*) [6]. Transcription factors have been reported to regulate the expression of structural genes, and the MYB-bHLH-WD40 (MBW) transcriptional complex is the main regulator involved in flavonoid biosynthesis [7]. Moreover, other transcription factors were also proven to participate in regulating anthocyanin biosynthesis, such as WRKY, bZIP, and ERF [8,9,10].

Eggplant (*Solanum melongena* L.) belongs to the Solanaceae family and is an important vegetable crop worldwide [6,11]. Eggplant fruits are widely consumed due to their generous composition of nutraceuticals [12]. The fruit peel color of eggplant has a wide range and mainly includes white, green, reddish purple, and dark purple, which is one of the primary exterior quality and economic traits. The commercial value of eggplant peel color has strong regional characteristics, and consumers in different regions have different preferences for it, especially in China. The color of eggplant fruit peel is mainly determined by the composition and contents of anthocyanins and chlorophyll [6]. The breeding of the full range of possible fruit colors is valued in diverse world markets [13].

Although many studies have been conducted on the fruit color of eggplant, most of them have mainly focused on purple fruit peel and light-induced anthocyanin biosynthesis [14,15,16]. The metabolic pathways and candidate genes that contribute to fruit peel coloration are still not fully understood. In addition, there are few reports on the non-anthocyanin flavonoid composition of eggplants with different fruit peel colors. Recently, the combined analyses of the transcriptome and metabolome have been widely used and have become a powerful tool to identify key genes and metabolites related to phenotypic traits, such as color variation and quality formation, in many horticultural plants [17,18]. Thus, the characteristic metabolic components and candidate regulatory genes underlying fruit peel coloration were investigated by integrated metabolome and transcriptome analyses with five different color varieties of eggplant in the present study. The regulatory networks and some new key genes involved in flavonoid synthesis were obtained through a weighted gene coexpression network analysis (WGCNA). The findings not only provide new insights into the molecular network of flavonoid synthesis but also provide valuable information for the future molecular breeding of eggplant.

## 2. Results

### 2.1. Variation in Fruit Peel Color and Total Anthocyanin and Chlorophyll Contents

**Figure 1:**
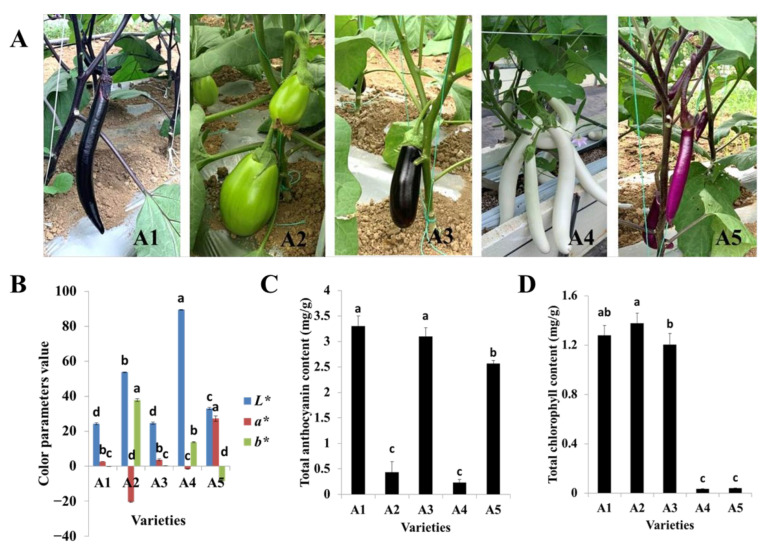
Phenotypes of the five eggplant varieties with different fruit colors. (**A**) Morphologies of the fruits of the five eggplant varieties; (**B**) Color parameters of fruit peels of the five eggplant varieties; (**C**) Total anthocyanin contents of the five eggplant varieties; (**D**) Total chlorophyll contents of the five eggplant varieties. The different letters on top of each bar indicate significant differences among the five eggplant varieties at *p* < 0.05.

The fruit colors of the five varieties used in this study were representative of the Chinese varieties, with A1 and A3 possessing black-purple fruit peels but different calyx colors, A2 possessing a green peel, A4 possessing a white peel, and A5 possessing a reddish-purple fruit peel (Figure 1A). The lightness values (L*) for A1 (black-purple), A2 (green), A3 (black-purple), A4 (white), and A5 (reddish-purple) were 24.21, 53.75, 24.61, 89.47, and 33.05, respectively. The black-purple cultivars A1 and A3 had the lowest lightness values. In other words, the fruit peel colors of A1 and A3 were deeper than those of the other varieties. Meanwhile, the redness values (a*) ranged from −20.54 to 27.23, and A5, with a reddish-purple fruit peel, exhibited the highest redness value. The yellowness parameters (b*) ranged from −8.55 to 37.88, and A2, with a green peel, possessed the highest yellowness value (Figure 1B).

The total anthocyanin and chlorophyll contents were also measured for the five varieties. The total anthocyanin content was highest in A1, followed by A3 and A5, while the total contents of anthocyanin in A2 and A4 were significantly lower (Figure 1C). The total chlorophyll content of A2 was slightly higher than those of A1 and A3. Very low amounts of total chlorophyll were detected in A4 and A5 (Figure 1D). Both total the anthocyanin and chlorophyll contents were lowest in A4. In general, the color values and pigment contents were in line with the fruit peel color variation.

### 2.2. Flavonoid Metabolic Differences

**Figure 2:**
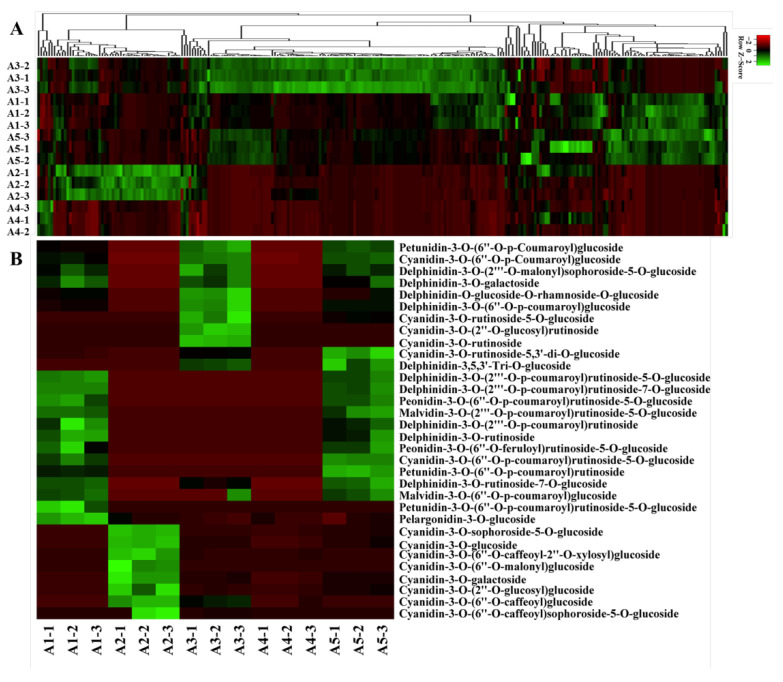
Metabolome analyses of different-colored eggplant fruits. (**A**) Hierarchical clustering of flavonoid metabolites in the five eggplant varieties; (**B**) The anthocyanins detected in the five eggplant varieties.

A widely targeted metabolite analysis was performed for the comprehensive profiling of flavonoids in colored eggplant fruit based on LC-MS/MS. A total of 260 flavonoid metabolites were identified, including 32 anthocyanins, 10 chalcones, 5 flavanols, 25 flavnones, 11 flavanonols, 58 flavones, 11 flavonoid carbonoside, 86 flavonols, 10 isoflavones, proanthocyanidin A6, and 11 tannins. A hierarchical cluster analysis (HCA) was carried out to demonstrate the phenotypic variations in terms of the relative contents of the five eggplant cultivars (Figure 2A).

A total of 32 anthocyanins were identified, including 10 delphinidin glycosides, 14 cyanidin glycosides, 3 petunidin glycosides, 2 malvidin glycosides, 2 peonidin glycosides, and 1 pelargonidin glycoside. The relative contents of the identified anthocyanins varied greatly in the five eggplant cultivars (Figure 2B). Delphinidin glycosides, petunidin glycosides, and malvidin glycosides were not detected in A2 and A4 with green and white fruit peels. Among the identified anthocyanins in the reddish to dark purple group (A1, A3, and A5), del-3-O-rutinoside (D3R), del-3-O-(2-O-p-coumaroyl)rutinoside-7-O-glu, and del-3-O-(2-O-p-coumaroyl)rutinoside-5-O-glu (nasunin) were present at the highest levels in A1 and A5. Interestingly, D3R and nasunin, which were previously reported to be the most representative anthocyanin compounds in purple eggplant, were not detected in A3, whereas the levels of del-3-O-(6-O-p-coumaroyl)glu and cya-3-O-(6-O-p-coumaroyl)glu were the highest in A3. In general, most of the identified anthocyanins were more abundant in A1, followed by A3 and A5.

In this study, remarkable differences in flavonoid metabolites were observed among the five varieties. Various flavonoid glycosides possessing rhamnose, galactose, rutinose, and glucose as major sugar ligands were detected. Kaempferol, quercetin, myricetin, luteolin, and their derivatives were the major flavonols and flavones present in the eggplants. The majority of the flavonoids accumulated at the lowest level in A4, followed by A2. As for the reddish to black-purple group (A1, A3, and A5), A1 and A5 seemed to have more similar flavonoid accumulation patterns compared to A3. Typically, most of the chalcones, tannins, and flavonols were more abundant in A3 than in A1 and A5.

**Figure 3:**
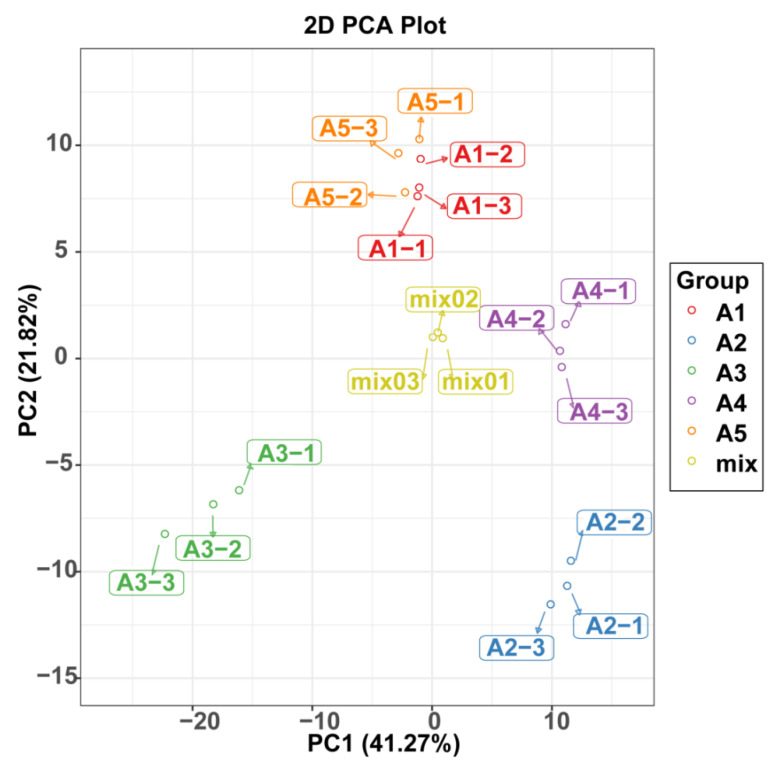
PCA score plot of flavonoid profiles from the five eggplant varieties.

A principal component analysis was also used to illustrate the internal structures of multiple variables in the five varieties (Figure 3). In the PCA plot, the quality control (QC) samples were grouped together, indicating the stability and repeatability of our analysis. Consistent with the HCA results, A1 and A5 showed the closet distance in the plot, indicating similarities in their metabolic accumulation pattern. Although A1 and A5 gathered near each other, the five varieties could be distinguished easily.

### 2.3. Comparison of Differentially Accumulated Metabolites among the Five Eggplant Varieties

**Figure 4:**
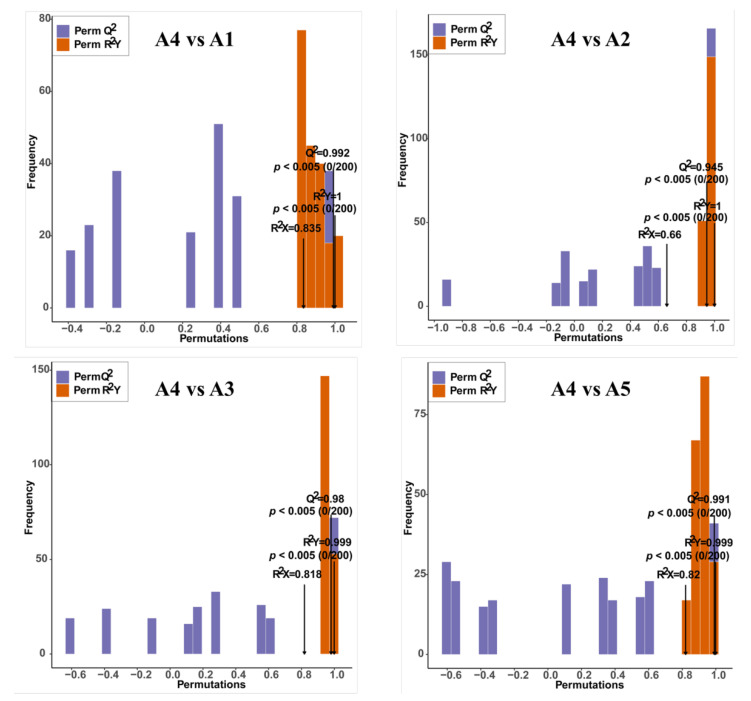
Orthogonal partial least-squares discriminant analysis (OPLS-DA) model of comparison groups, including A4 vs. A1, A4 vs. A2, A4 vs. A3, and A4 vs. A5.

Score plots of the orthogonal partial least-squares discriminant analysis (OPLS-DA) were created to analyze the differences between pairs of the five varieties. High predictability (Q2 > 0.9) and strong goodness of fit (R2X and R2Y) of the OPLS-DA models were observed in pairwise comparisons among the five varieties (Figure 4 and Supplementary Figure S1), suggesting the reliability of the metabolic data. Although in the PCA plots A1and A5 gathered near each other, in the OPLS-DA models, A1, A2, A3, A4, and A5 were clearly separated from each other, suggesting major distinctions in the flavonoid metabolic profiles between the different peel color groups.

**Figure 5:**
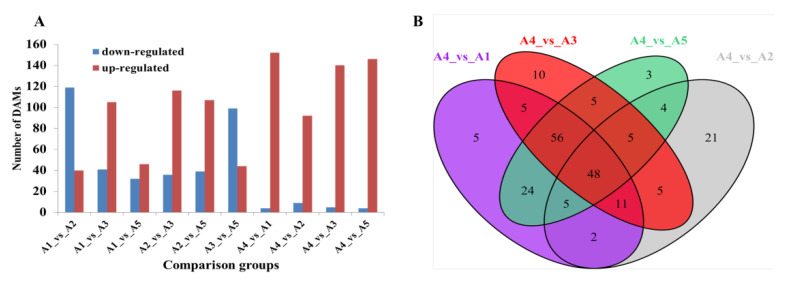
Differential flavonoid analysis among the five eggplant varieties. (**A**) Number of differentially accumulated flavonoids among the five eggplant varieties; (**B**) Venn diagram showing the overlapping and cultivar-specific differential metabolites among the comparison groups, including A4 vs. A1, A4 vs. A2, A4 vs. A3, and A4 vs. A5.

The differentially accumulated metabolites (DAMs) were further identified in pairwise comparisons using the criteria of a fold change ≥2 or ≤0.5 and a VIP (variable importance in project) ≥1. Compared with A4, possessing a white fruit peel color, a total of 156, 101, 145, and 150 DAMs were identified in A1 (black purple), A2 (green), A3 (black purple), and A5 (reddish purple), respectively (Figure 5A). As expected, most of the DAMs were upregulated in the other four varieties as the peel color changed from white to black purple. A total of 48 DAMs were observed among the comparison groups A4 vs. A1, A4 vs. A2, A4 vs. A3, and A4 vs. A5 (Figure 5B).

For the reddish to black-purple group (A1 vs. A3, A1 vs. A5, and A3 vs. A5), the lowest number of DAMs was found for A1 vs. A5 (46 upregulated and 32 downregulated DAMs in A5). Compared with A3, a total of 146 DAMs (105 downregulated and 41 upregulated) as well as 143 DAMs (99 downregulated and 44 upregulated) were observed in A1 and A5, respectively (Figure 5A). Most of the DAMs, including chalcones, flavanonols, flavonols, and tannins, were upregulated in A3.

The differential metabolites among the five varieties were mapped to the Kyoto Encyclopedia of Genes and Genomes (KEGG) database, and a KEGG enrichment analysis was performed for the DAMs of each comparison group. The most enriched pathways detected for all compared groups were flavonoid biosynthesis, anthocyanin biosynthesis, flavone and flavonol biosynthesis, isoflavonoid biosynthesis, and the biosynthesis of secondary metabolites.

### 2.4. Comparative Transcriptome of Fruit Peels among the Five Eggplant Varieties

A total of fifteen libraries were established using the five varieties of samples with three biological replicates for each variety. After removing the adaptor and low-quality sequences, a total of 20.66~21.4 Gb of clean reads were obtained for each variety. The Q20 and Q30 values for each library were equal to or greater than 97.43 and 92.92%, respectively (Table 1). These clean reads were mapped to the reference genome with match ratios in the range of 96.64~97.91 %. Differentially expressed genes (DEGs) were identified with an expression fold change of log_2_ ratio ≥ 1 and a false discovery rate ≤ 0.01 as the thresholds, and a total of 10110 DEGs were obtained among all samples (Supplementary Table S1). There were 4265, 3987, 4005, 4253, 3837, 3629, 4456, 4095, 2881, and 2152 DEGs in the comparison groups, respectively, including A1 vs. A2, A1 vs. A3, A1 vs. A5, A2 vs. A3, A2 vs. A5, A3 vs. A5, A4 vs. A1, A4 vs. A2, A4 vs. A3, and A4 vs. A5 (Table 2). Among these comparison groups, the A4 vs. A5 group had the smallest number of upregulated and downregulated DEGs.

A GO enrichment analysis of these DEGs showed that the genes related to “cell”, “cell part”, and “organelle” were predominant in the cellular component category. In the molecular function category, the most enriched terms were binding and catalytic activity. In the biological process category, the genes were mostly involved in metabolic and cellular processes. For the KEGG annotation results, the DEGs among all samples were mapped to 141 KEGG pathways, and the KEGG pathways of the biosynthesis of secondary metabolites, plant hormone signal transduction, phenylpropanoid biosynthesis, flavonoid biosynthesis, anthocyanin biosynthesis, photosynthesis, photosynthesis-antenna proteins, and porphyrin and chlorophyll metabolism were significantly enriched.

### 2.5. Identification of DEGs Involved in Flavonoid Biosynthesis

**Figure 6:**
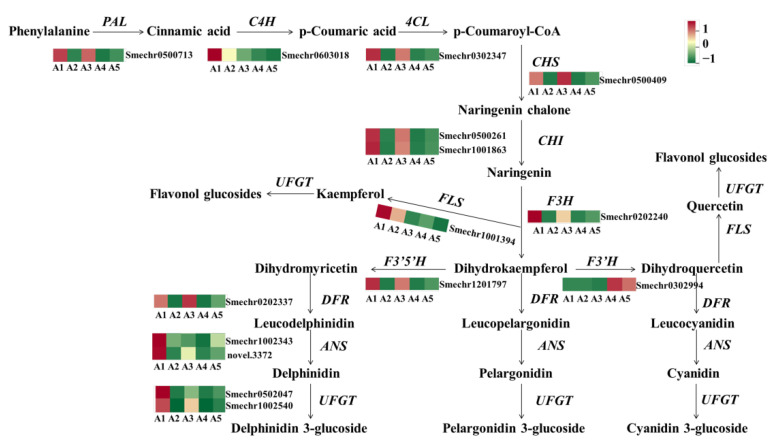
The expression profiles of genes involved in the anthocyanin/flavonoid pathway. PAL, phenylalanine ammonia lyase; C4H, cinnamate 4-hydroxylase; 4CL, 4-coumarate-CoA ligase; CHS, chalcone synthase; CHI, chalcone isomerase; F3H, flavanone 3-hydroxyl enzyme; FLS, flavonol synthase; DFR, dihydroflavonol reductase; ANS, anthocyanin synthase; UFGT, flavonoid 3-O-glucosyltransferase.

To explore the mechanism of accumulating flavonoids in the different fruit peel colors of eggplant, the gene expression patterns involved in the phenylpropanoid and flavonoid pathways were analyzed. As expected, most of the structural genes involved in flavonoid biosynthesis, including *PAL*, *C4H*, *4CL*, *CHS*, *CHI*, *F3H*, *DFR*, *ANS*, and *UFGT* had significantly lower levels of transcription in A2 and A4, which may be one of the reasons for lower flavonoids in A2 and A4 (Figure 6). *F3. wH* was found to have no or very weak expression levels in A2 and A4, suggesting that *F3thaH* may be a critical factor that directly determines the purple color of fruit peels in eggplant.

The structural genes involved in the flavonoid biosynthesis pathway were also differently expressed among the comparison groups of A1 vs. A3, A1 vs. A5, and A3 vs. A5. In the comparisons of A1 vs. A5 and A3 vs. A5, both early and late biosynthesis genes were downregulated in A5, including *PAL*, *4CL*, *C4H*, *CHS*, *CHI*, *F3H*, *F3 3HH*, *DFR*, *ANS*, and *UFGT*. Notably, in the A1 vs. A3 comparison, the early biosynthesis genes, including *CHS*, *CHI*, *F3H*, and *F3nd H*, were not differently expressed, and most of the DEGs were late biosynthesis genes, such as *DFR*, *ANS*, and anthocyanin-modified genes (Smechr0502047, Smechr0400421, and Smechr1102789).

In general, most of the structural genes involved in flavonoid biosynthesis were highly expressed in A1 and A3 with black-purple fruit peels. With decreasing purple color, the expression of most annotated flavonoid genes decreased in A5, while the lowest expression levels were found in A2 and A4.

### 2.6. Identification of DEGs Involved in Chlorophyll Metabolism

**Figure 7:**
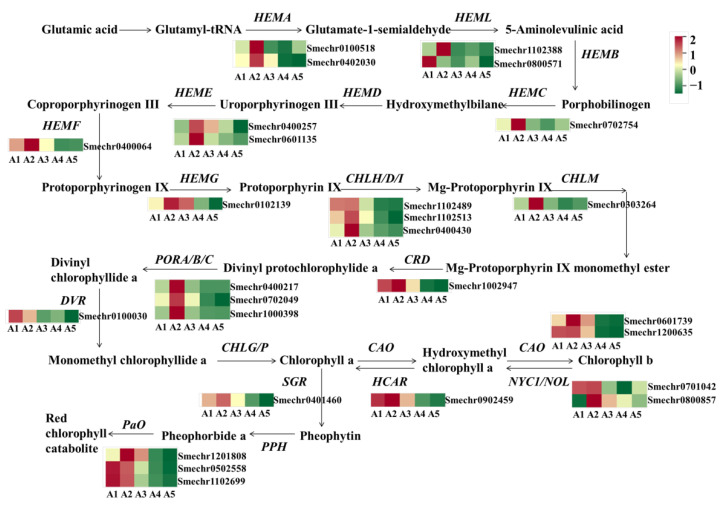
Differential expression of genes related to the chlorophyll metabolism pathway. HEMA, Glutamyl-RNA reductase; HEMB, Delta-aminolevulinic acid dehydratase; HEMC, Porphobilinogen deaminase; HEME, Uroporphyrinogen decarboxylase; HEMF, Coproporphyrinogen-III oxidase; HEMG, Protoporphyrinogen III oxidase; CHLH/D/I, Magnesium-chelatase subunit ChlH/ChlD/ChlI; CHLM, Magnesium protoporphyrin IX methyltransferase; CRD, Magnesium-protoporphyrin IX monomethyl ester cyclase; DVR, Diviyl chlorophyllide a 8-vinyl-reductase; POR, Protochlorophyllide oxidoreductase; CHLG, Chlorophyll synthase; NYC1/NOL: Chlorophyll (ide) b reductase; CAO, chlorophyllide a oxygenase; HCAR, 7-hydroxymethyl chlorophyll a reductase; SGR, Stay-green; PPH, Pheophytinase; PaO, Pheophorbide a oxygenase; RCCR, Red Chl catabolite reductase.

The green color of eggplant fruit peels is mainly affected by chlorophylls. In this study, a total of 27 DEGs related to chlorophyll metabolism were identified (Figure 7). Compared to A2 with green fruit peels, most of the DEGs showed lower expression in A4 and A5, including *HEMA* (Smechr0402030 and Smechr0100518), *HEMC* (Smechr0702754), *HEME* (Smechr0400257 and Smechr0601135), *HEMF* (Smechr0400064), *HEMG* (Smechr0102139), *CHLH* (Smechr0400430), *CHLM* (Smechr0303264), *CRD* (Smechr1002947), *POR* (Smechr0702049), *DVR* (Smechr0100030), *CAO* (Smechr0601739 and Smechr1200635), *NOL* (Smechr0701042), and *HCAR* (Smechr0902459).

As for the comparison of A2 vs. A1, only four DEGs related to chlorophyll biosynthesis were detected, while in A2 vs. A3 there were eight DEGs exhibiting higher expression levels in A2, including *HEMA*, *HEML*, *HEMC*, *CHLH*, *CHLM*, and *POR*. Notably, except for A2 vs. A1, the pathway of photosynthesis-antenna proteins was also enriched, based on a KEGG analysis of the comparison of A2 vs. A3, A2 vs. A4, and A2 vs. A5. A total of 15 DEGs related to the light-harvesting chlorophyll protein complex (LHC), including 7 DEGs related to LHCI and 8 DEGs related to LHCII, exhibited declined expression in A4 and A5 (Supplementary Table S2).

### 2.7. Identification of DEGs Involved in Hormone-Mediated Signaling Pathways

The KEGG enrichment analysis showed that the plant hormone signal transduction pathway was one of the most significantly enriched pathways. Therefore, to better investigate hormonal regulation in fruit peel color variation, the DEGs enriched in the signaling pathways of auxin, gibberellin, ethylene, brassinosteroid (BR), abscisic acid (ABA), salicylic acid (SA), and jasmonic acid (JA) were analyzed (Supplementary Figure S2).

In the auxin signaling pathway, the expression levels of five DEGs were correlated with color variation, including *AUX/IAA* (Smechr0303465), *ARF* (Smechr0101326), *GH3* (Smechr0400187), and *SAUR* (Smechr0100144 and Smechr1100127). In the gibberellin signaling pathway, the expression of *GID1* (Smechr1000150) and *DELLA* (Smechr0500162 and Smechr0500354) exhibited positive correlations with fruit peel color variation. Similarly, the expression of *ETR* (Smechr0902323) and *EBF1/2* (Smechr0700262 and Smechr1200241), which are involved in the ethylene signaling pathway, as well as *BRI1* (Smechr0702406) and *BKI1* (Smechr0502339), which are involved in the BR signaling pathway, were associated with color variation among the five varieties. *ABF* (Smechr0100651) and *SnRK2* (Smechr0401004 and Smechr0802058) in the ABA signaling pathway showed higher expression levels in A1 and A3 with black-purple fruit skins. Meanwhile, the expression of *TGA* (Smechr1002700) in the SA signaling pathway was correlated with color variation among the five varieties. Specifically, two bHLH transcription factors involved in the JA signaling pathway had different expression levels among the five varieties, of which *SmTT8* (Smechr0901701) was also involved in flavonoid biosynthesis.

### 2.8. Identification of Coexpressed Gene Networks and Key Candidates

**Figure 8:**
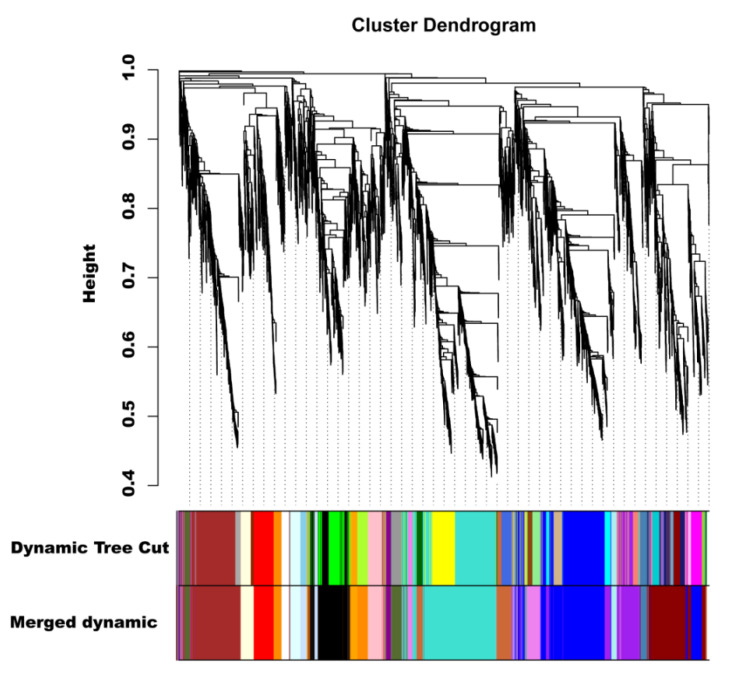
Hierarchical clustering presenting twenty modules with coexpressed genes.

**Figure 9:**
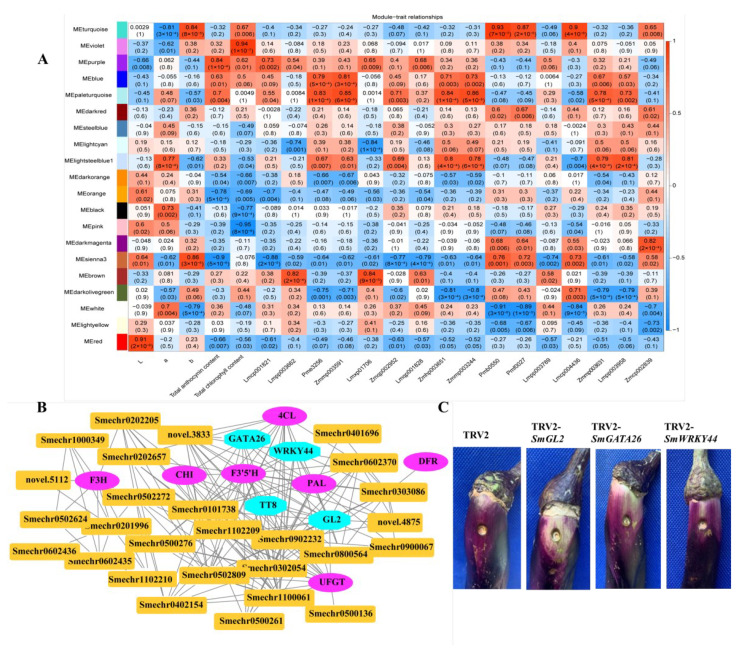
Gene network and key candidate genes involved in anthocyanin biosynthesis, identified by WGCNA. (**A**) Module–trait associations based on Pearson correlations; (**B**) Gene network of the hub genes in the purple module, which were positively correlated with the total anthocyanin content (*r^2^* = 0.84, *p* = 1× 10^−4^); (**C**) The effect of *SmGL2*, *SmGATA26,* and *SmWRKY44* silencing in VIGS eggplant fruits.

To investigate the gene regulatory network of peel coloration of eggplant fruit, a weighted gene coexpression network analysis (WGCNA) using 6241 non-redundant DEGs was performed, and 20 distinct modules that were labeled in different colors were obtained (Figure 8). The total anthocyanin contents and total chlorophyll contents as well as delphinidin-type anthocyanins were used as phenotypic data for the analysis. The analysis revealed that the purple and violet modules had positive correlations with the anthocyanin contents and chlorophyll contents, respectively (Figure 9A). The KEGG enrichment analysis of genes grouped into different modules showed that genes in the purple module were enriched in flavonoid biosynthesis, while genes in the violet module were enriched in photosynthesis-antenna proteins and porphyrin and chlorophyll metabolism.

In the correlation network of the purple module, four transcription factors, including *SmGL2* (Smechr0303487), *SmGATA26* (Smechr0601814), *SmWRKY44* (Smechr1002420), and *SmTT8* (Smechr0901701) as well as genes involved in the flavonoid biosynthesis pathway, such as *SmF3mFtH* (Smechr1201797), *SmUFGT* (Smechr1002540), *SmPAL* (Smechr0500713), and *Sm4CL* (Smechr0302347), which had the highest degree of connectivity, were identified as hub genes (Table 3, Figure 9B). SmWRKY44 and SmTT8 were previously reported to be involved in anthocyanin biosynthesis. Thus, to further investigate the function of SmGL2 and SmGATA26, the TRV2-*SmGL2*, TRV2-*SmGATA26*, and TRV2-*SmWRKY44* recombinants were constructed to silence *SmGL2*, *SmGATA26*, and *SmWRKY44* in the purple peels of eggplants. Compared to TRV2, the *SmGL2*-silenced, *SmGATA26*-silenced, and *SmWRKY44*-silenced eggplants had decreased anthocyanin accumulation in the fruit peels, suggesting that SmGL2 and SmGATA26 may be involved in anthocyanin biosynthesis (Figure 9C).

In the correlation network of the violet module, two genes belonged to the LHCB family, including *SmLhcb3* (Smechr1000379) and *SmLhcb4* (Smechr0900664) as well as *SmTIC55* (Smechr0502558) and *SmCAO* (Smechr1200635), which are involved in porphyrin and chlorophyll metabolism, had the highest degree of connectivity and were identified as hub genes (Supplementary Figure S3).

### 2.9. Validation of Differentially Expressed Genes by Quantitative Real-Time PCR

**Figure 10:**
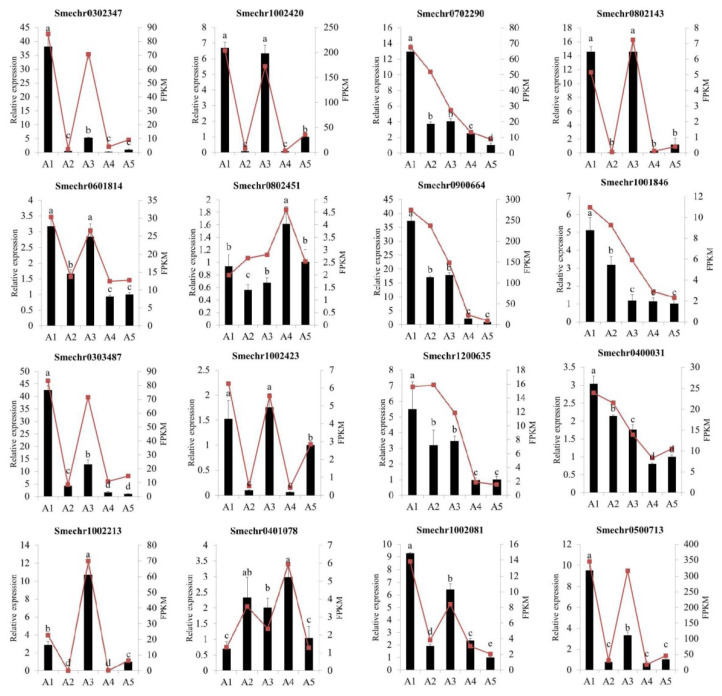
Quantitative real-time PCR analysis of the sixteen DEGs among the five eggplant cultivars. The different letters on top of each bar indicate significant differences among the five eggplant varieties at *p* < 0.05.

To validate the accuracy of the RNA-Seq data, a total of 16 differentially expressed genes, including 8 hub genes, were selected for a quantitative real-time PCR (qRT-PCR) analysis. As illustrated in Figure 10, the qRT-PCR results showed that the expression patterns of these genes were consistent with the RNA-seq results, indicating that the transcriptome sequencing results were reliable.

## 3. Discussion

### 3.1. Pigment Accumulation in Different Colors of Eggplant Fruit Peels

Anthocyanins were recognized as the major pigments in red to black-purple eggplant fruit peels. Previous studies suggested that delphinidin-3-O-(2-O-p-coumaroyl)rutinoside-5-O-glucoside (nasunin) and delphinidin-3-O-rutinoside (D3R) were the main types of anthocyanins in the Japanese-type and non-Japanese-type eggplants, respectively [19,20]. In our study, delphinidin and cyanidin derivatives were the major detected anthocyanins, and delphinidin was the most abundant. Cyanidin derivatives, but not delphinidin derivatives, were identified in A2 and A4, which supported that delphindin-type anthocyanins were more responsible for the red to black-purple pigmentation of eggplant fruit peels. Interestingly, both nasunin and D3R were highly accumulated in A1 and A5 but not in A3, suggesting that the types of anthocyanins are genotype-dependent. This variation may be due to the different origins and genetic backgrounds, as A3 belongs to the European type of eggplant, exhibiting more adaption to low temperatures, while A1 and A5 belong to the Chinese type, which have better fruit taste.

Previous studies have suggested that flavonoid metabolites, including tannins, flavones, and flavonols, are important copigment factors, contributing to the color of most flowers, fruits, and seeds [4,5]. The hydrophobic interactions between anthocyanin and other organic components form molecular associations and stabilize their final color appearance [21]. In this study, higher levels of tannins and flavonol glycosides were detected in the reddish to black-purple group, especially in A3. Despite no significant differences being found in the appearance of fruit peels and the total anthocyanin contents between A1 and A3, big differences in the compositions and contents of flavonoid metabolites still existed, suggesting that the pigment accumulation in eggplant fruit peels is a complex process and that the final purple color appearance can be attributed to a combination of multiple factors.

It has been reported that the presence of both anthocyanins and chlorophylls resulted in a black-purple fruit color, and dark purple fruits had higher levels of chlorophyll than dark-green ones [19]. However, the total content of chlorophyll of A2 was slightly higher than those of A1 and A3, but the total chlorophyll contents of A1, A2, and A3 did not vary significantly. The presence of chlorophylls may have a minor darkening effect on the black-purple appearance [6].

### 3.2. The Activation of Early Phenylpropanoid/Flavonoid Biosynthesis Genes for Anthocyanin Accumulation in Fruit Peels

Anthocyanins are produced by a branch of the phenylpropanoid and flavonoid pathway, and differences in the expression pattern of genes involved in phenylpropanoid/flavonoid biosynthesis result in diverse anthocyanin profiles [22]. Our study provides a comprehensive profile of the flavonoid biosynthesis pathway of eggplant fruit peels with different colors. The results revealed by the WGCNA showed that several structural genes, including *SmPAL*, *Sm4CL*, *SmF3mFCH*, and *SmUFGT* were identified as hub genes associated with anthocyanin accumulation. In the initial steps of the flavonoid pathway, enzymes encoded by *PAL*, *C4H*, and *4CL* sequentially catalyze the metabolism of phenylalanine to coumaroyl-CoA, which provides the precursor compounds for the synthesis of flavonoids [2]. Considering that all of the structural genes involved in anthocyanin biosynthesis had higher expression in A1 and A3, and the higher expression of *SmPAL*, *SmC4H*, and *Sm4CL* provided sufficient precursor compounds for the synthesis of flavonoids, we concluded that the activation of early phenylpropanoid/flavonoid biosynthesis genes was more responsible for anthocyanin accumulation in eggplant.

F3′5′H is an important enzyme that catalyzes the hydroxylation of flavonoids at C3′ and C5′ of ring B, resulting in the production of delphinidin-based anthocyanins [23]. In this study, undetectable or very low expression of *SmF3mr H* were observed in A2 and A4 with green and white fruit peel colors, but high expression was found in A1 and A3, followed by A5. Thus, we concluded that *SmF3mFuH* was the key factor for the purple color formation of fruit peels at the transcriptional level. This was consistent with a recent report by Yang et al. [24]. Another key gene was anthocyanin 3-O glucose transferase (*UFGT*), which is the key enzyme for anthocyanin stability and water solubility [2]. Previous studies showed that *UFGT* was the key gene involved in the accumulation of anthocyanins in jujube fruit peels [25,26]. *UFGT* was also found to be present but not expressed in white skin apples [27]. Their findings were in line with our results that the different expression levels of *SmUFGT* coincided with the different anthocyanin accumulations and colorations among the five eggplant cultivars.

### 3.3. Candidate Transcription Factors Modulated Anthocyanin Accumulation in Eggplant Fruit Peels

The MYB-bHLH-WD40 transcriptional complex is considered to be an important and conserved model in the regulation of anthocyanin biosynthesis [7]. In eggplant, previous studies have reported that several MYB and bHLH TFs, including SmMYB113, SmMYB86, SmMYB75, SmMYB35, SmbHLH1, SmbHLH13, and SmTT8, are involved in anthocyanin biosynthesis, of which SmMYB113 and SmTT8 were proven to be the key factors responsible for anthocyanin accumulation [24,28,29,30,31,32,33]. In our study, very low transcription levels of *SmMYB113* were detected in A2 and A4, and compared to the reddish to black-purple group (A1, A3, and A5), a 6 bp deletion in the first exon of *SmMYB113* was found in white and green fruit peels of eggplants (Supplementary Figure S4). A previous report on grapes revealed that a retrotransposon-induced mutation in *VvmybA1* is associated with the loss of pigmentation in white cultivars [34]. Whether the different gene expression levels or the difference in sequence variation in *SmMYB113* affects anthocyanin accumulation in white and green eggplant fruit peels needs further investigation. Interestingly, although SmMYB113 and SmTT8 were proven to participate in the regulation of anthocyanin biosynthesis, only the transcript levels of *SmTT8* correlated with those of anthocyanin biosynthesis structural genes among different-colored fruits. Additionally, four transcription factors, including *SmGL2*, *SmGATA26*, *SmWRKY44*, and *SmTT8*, were also considered as hub genes associated with anthocyanin accumulation based on the WGCNA analysis. Thus, we speculated that SmTT8 is the backbone of the anthocyanin biosynthetic regulation network. This finding was in line with a previous study on mulberry fruit colors, which showed that bHLH3 is indispensable for the regulation of the flavonoid pathway [3]. However, the reason for the differences in the expression levels of *SmTT8* among different-colored fruits remains unknown.

In addition to the MYB-bHLH-WD40 regulatory complex, which is involved in anthocyanin biosynthesis, many transcription factors, such as WRKY, NAC, and HD-ZIP, have been reported to regulate anthocyanin biosynthesis [8,9,35]. Most of them exert their function either by regulating the expression of the MBW complex components at the transcriptional level or by destabilizing the MBW complex through protein–protein interactions [36]. The different expression levels of *SmTT8* among different-colored fruit peels may be caused by other TFs, such as SmGL2, SmGATA26, or SmWRKY44. SmGL2 is a homolog of Arabidopsis GL2. In Arabidopsis, AtGL2 is a negative regulator of anthocyanin synthesis that directly binds the TAAATGTT/A L1 box in the promoters of *AtPAP2* and *AtMYB113*, repressing their expression at the transcriptional level [37]. SmWRKY44 was previously reported as a hub gene involved in light-induced anthocyanin biosynthesis in eggplant [38]. The transcript levels of *SmGL2*, *SmGATA26*, and *SmWRKY44* were positively correlated with the expression of *SmTT8* in the connection network. These genes may have upstream or downstream regulatory relationships with SmTT8. In addition, the TRV2-*SmGL2* and TRV2-*SmGATA26* silencing vector effectively inhibited anthocyanin accumulation in fruit peels, indicating that SmGL2 and SmGATA26 are involved in anthocyanin biosynthesis. The exact functions of SmGL2 and SmGATA26 in the anthocyanin biosynthetic regulation network remain unclear and will be the focus of future research. Nevertheless, the hub genes and regulation network presented in our study broaden our understanding of anthocyanin biosynthesis in eggplant.

### 3.4. Plant Hormone Signaling Pathway for Color Variation in Eggplant Fruit Skins

Phytohormones play important roles in regulating anthocyanin accumulation [39]. However, few studies have been reported on eggplant. In the present study, plant hormone signal transduction was one of the most significantly enriched pathways. The signaling pathway from hormone perception to anthocyanin regulation is relatively clear for jasmonate, auxin, strigolactone, and gibberellic acid [36]. The basic logic of the signaling mechanisms for these four hormones is similar. They all involve an Skp1/Cullin/F-box (SCF) E3 ubiquitin ligase complex that targets a protein substrate for poly-ubiquitination and degradation by the 26S proteasome [36]. In Arabidopsis, JAZ proteins directly bound the MYB and bHLH TFs of the anthocyanin-activating MBW complex to regulate jasmonate-mediated anthocyanin accumulation [40]. In apples, MdJAZ1 interacted with MdTRB1 and interfered with the interaction between MdTRB1 and MdMYB9, thereby negatively modulating MdTRB1-promoted anthocyanin biosynthesis [41]. In this study, several bHLH TFs, including SmTT8 in the JA signaling pathway, were also found to be differently expressed among different-colored fruits, indicating a possible involvement of JA-mediated regulation of anthocyanin biosynthesis. In addition, some of the genes involved in the phytohormone signaling pathway, such as ethylene, abscisic acid, salicylic acid, and BR, also exhibited different expression levels correlated with color variation in this study. Whether these hormones have a positive or negative effect on anthocyanin accumulation seems to vary from species to species [36]. In apples, MdERF1B bound to the promoters of *MdMYB9* and *MdMYB11* and regulated anthocyanin and proanthocyanidin accumulation [42]. ABI5 regulated ABA-induced anthocyanin biosynthesis by modulating the MdMYB1-MdbHLH3 complex [43]. However, the signaling mechanisms that regulate anthocyanin biosynthesis are still poorly understood. The crosstalk of these signaling pathways might be extensive to respond to different developmental and environmental cues. The elucidation of the molecular nature of these interactions will be valuable for augmenting the understanding of the regulation of anthocyanin biosynthesis.

## 4. Materials and Methods

### 4.1. Plant Materials

Five eggplant varieties, A1 (cv. Suqie 6), A2 (cv. Suqie 801), A3 (cv. Bulita), A4 (Suqie 11), and A5 (cv. Hangqie 1), with different fruit peel colors were used in this study. The fruit peel color of A1 and A3 is black-purple. A1 is the Chinese type with long fruit and a purple calyx, while A3 is the European type with ovate fruit and a green calyx. A2, A4, and A5 are all Chinese types, and their fruit peel colors are green, white, and reddish purple, respectively. The seeds of A1, A2, and A4 were collected from Jiangsu Academy of Agricultural Sciences, while the seeds of A3 and A5 were bought from a Chinese market. All of the samples were grown in a completely randomized block design in a greenhouse at the Liuhe experimental station (32°29′17″N and 118°37′17″E) of Jiangsu Academy of Agricultural Sciences from March to August 2021. 

The fruit peels of the five varieties were collected at the commercial stage (30 days after anthesis). Six fruit peels from six individual plants were pooled as a sample, and three samples were set as biological replicates for each variety. Before the fruit peels were collected, the fruit peel color intensity was measured for each variety. The fruit peel samples of each cultivar were divided into three sample sets and stored at −80 °C for subsequent analysis. One sample set was used for total anthcoyanin and chlorophyll content measurement, and the other two sample sets were used for metabolite analysis and transcriptome sequencing (qRT-PCR), respectively. 

### 4.2. Total Anthocyanin and Chlorophyll Analysis

The total anthocyanin contents of the five varieties were measured using the spectrophotometric differential pH method [6]. First, 100 mg samples were ground into powder and extracted with 2 mL of a pH 1.0 buffer (containing 50 mM KCl and 150 mM HCl) and a pH 4.5 buffer (containing 400 mM sodium acetate and 240 mM HCl). The extraction solutions were vortexed and stored at 4 °C for 12 h. The solutions were then centrifuged at 10,625× *g* for 20 min at 4 °C. The absorption at 510 nm was measured, and the total anthocyanin content was calculated according to Zhang et al. [44]. The extraction of chlorophyll of the five varieties was performed as previously described [6]. Approximately 200 mg samples were ground into powder and extracted with 5 mL of 80% acetone. The extraction was then centrifuged at 3000× *g* for 20 min. The supernatants were collected and measured at the absorption wavelengths of 663 nm and 645 nm. 

### 4.3. Fruit Peel Color Intensity Measurement

The fruit peel colors of the five varieties were analyzed as previously described [6]. The values of L* (lightness), a* (redness and greenness), and b* (yellowness and blueness) were measured using a handheld spectrophotometer (CR-400, Minolta, Japan). The L* value represents the fruit peel color intensity, and a lower L* value generally means a deeper color. 

### 4.4. Flavonoid Identification and Quantification

The sample preparation and flavonoid identification and quantification were performed at Wuhan MetWare Biotechnology Co., Ltd. (www.metware.cn, accessed on 12 June 2021) following the standard procedures. The sample extracts were analyzed using an LC-ECI-MS/MS system (UPLC, SHIMADZU Nexera X2, www.shimadzu.com.cn, accessed on 12 June 2021; MS, Applied Biosystems 4500 Q TRAP, www.appliedbiosystems.com.cn, accessed on 12 June 2021). Normalized metabolite data from the five varieties were used to compare the metabolites. A hierarchical clustering analysis (HCA), a principal component analysis (PCA), and an orthogonal partial least-squares discriminant analysis (OPLS-DA) were processed by the R-package to study the metabolite differences among the five samples. The OPLS-DA model plots were created using the MetaboAnalystR (v1.0.1) package. The significantly regulated metabolites between groups were determined by the criteria of VIP ≥ 1 and a fold change ≥2 or ≤0.5. The Kyoto Encyclopedia of Genes and Genomes (KEGG) compound database (http://www.kegg.jp/kegg/compound, accessed on 12 June 2021) was used for the annotation of the identified metabolites, and annotated metabolites were then mapped to the KEGG pathway database (http://www.kegg.jp/kegg/pathway.html, accessed on 12 June 2021).

### 4.5. RNA-Seq Analysis

The total RNA was extracted from fruit peels of the five varieties using the RNeasy Plant Mini Kit (Qiagen, Hilden, Germany). The RNA quantity, integrity, and purity were analyzed using a Nanodrop and an Agilent Bioanalyzer 2100 (Agilent Technologies, Palo Alto, CA, USA). The sequencing of the libraries was performed on an Illumina HiSeq platform. The clean reads for each library were 6.38–7.45 Gb after filtration. All clean reads were then mapped to the eggplant reference genome (http://eggplant-hq.cn, accessed on 21 June 2021), with match ratios in the range of 96.64–97.91%.

### 4.6. Bioinformatic Analysis

FPKM (fragments per kb per million fragments) was used for gene expression level quantification [45]. Differentially expressed genes (DEGs) were identified by DESeq2 [46]. The threshold of the *p* value was calculated using the false discovery rate (FDR) control method. The criterion for the judgment of significance of the DEGs was set at an FDR < 0.01 and the absolute value of log_2_ ratio ≥ 1. The gene ontology (GO) and KEGG pathway enrichment analyses were performed using the topGO method and KOBAS 2.0 (http://www.biostars.org/p/200126, accessed on 12 June 2021), respectively [47].

### 4.7. Coexpression Network Analysis 

A weighted gene coexpression network analysis (WGCNA) was performed for all differentially expressed genes using the WGCNA R package [48]. The adjacency matrix was generated by calculating the Pearson correlations among all the genes. A correlation analysis between the identified modules and sample traits was performed with all genes in each module. The hub genes related to the total anthocyanin content and chlorophyll content in the significant positive module were used to construct coexpression networks with Cytoscape 3.7.2 (http://www.cytoscape.org, accessed on 12 June 2021).

### 4.8. Quantitative Real-Time PCR Analysis

The quantitative reverse-transcription PCR (qRT-PCR) was applied to analyze the expression levels of 16 DEGs among the five varieties. First-strand cDNA was syntheized by the ReverTran Ace qPCR RT kit (Toyobo). The primers used for qRT-PCR were designed by Primer 3 (http://primer3.ut.ee, accessed on 12 June 2021). All primers are listed in Supplementary Table S3. qRT-PCR was performed as previously described [6]. The gene expression levels were normalized with *SmGAPDH* as an internal control and were calculated using the 2^−ΔΔCt^ method [49].

### 4.9. Vector Construction and Transformation of Eggplant

The tobacco rattle virus (TRV)-based VIGS system was employed for SmGL2, SmGATA26, and SmWRKY44 silencing. The 300 bp best target regions of SmGL2, SmGATA26, and SmWRKY44 predicted by SGN VIGS Tool (https://vigs.solgenomics.net, accessed on 12 June 2021) were cloned into a pTRV2 vector using specific primers. The TRV1, empty TRV2, TRV2-SmGL2, TRV2-SmGATA26, and TRV2-SmWRKY44 plasmids were then transformed into Agrobacterium tumefaciens strain GV3101. An equal volume of TRV1 Agrobacterium culture was mixed with empty TRV2 or TRV2-SmGL2, TRV2-SmGATA26, and TRV2-SmWRKY44 cultures before infiltration. The mixtures were then infiltrated into eggplant peels via needleless syringes. The treated eggplant fruits were kept in the dark for 24 h at 22 °C and then cultivated in regular light conditions for 5 days. Three biological replicates were conducted for each combination.

### 4.10. Statistical Analysis

The statistical analysis was performed using Microsoft Excel 2013 and SPSS statistics 19 (IBM SPSS, Chicago, IL, USA). Significant differences were determined using a one-way ANOVA with Duncan’s test at *p* < 0.05.

## 5. Conclusions

In this study, delphinidin-type anthocyanins were the main anthocyanin components in reddish to black-purple eggplant fruit peels. The activation of early phenylpropanoid biosynthesis genes was more responsible for anthocyanin accumulation in eggplant, while *SmF3mFnH* was the key factor for the formation of a purple color. Moreover, four TFs, including *SmGL2*, *SmGATA26*, *SmWRKY44*, and *SmTT8*, were identified as hub genes associated with anthocyanin accumulation. In addition, the pathway of plant hormone signal transduction was significantly enriched. It is possible that phytohormones cooperatively interact to modulate flavonoid biosynthesis. The results of this study provide valuable information and new insights into the regulatory network of fruit coloration and will therefore contribute to the molecular breeding of eggplant.

## Figures and Tables

**Table 1:** Overview of the transcriptome sequencing data and quality check.

Samples	Clean Reads	Reads Mapped	Q20 (%)	Q30 (%)	GC Content (%)
A1–1	47409416	45993726 (97.01%)	97.69	93.49	42.23
A1–2	46685604	45365789 (97.17%)	97.77	93.64	42.46
A1–3	48592694	47236677 (97.21%)	97.8	93.68	42.69
A2–1	48679876	47284281 (97.13%)	97.75	93.59	42.33
A2–2	43769020	42395918 (96.86%)	97.67	93.51	41.91
A2–3	49045732	47596182 (97.04)	97.68	93.41	42.18
A3–1	46608676	45126433 (96.82)	97.76	93.63	41.66
A3–2	45840606	44446440 (96.96%)	97.8	93.73	41.9
A3–3	49665704	47998296 (96.64%)	97.68	93.5	42.34
A4–1	47772226	46365463 (97.06%)	97.79	93.67	42.50
A4–2	42518600	41148256 (96.78%)	97.43	92.92	42.12
A4–3	49236290	47743262 (96.97%)	97.77	93.64	42.22
A5–1	46807752	45518684 (97.25%)	97.88	93.9	42.42
A5–2	47101278	46077478 (97.83%)	98.15	94.37	42.34
A5–3	43808476	42891632 (97.91%)	98.23	94.57	42.44

**Table 2:** The number of DEGs identified in the pairwise comparisons.

Comparison Groups	Total DEGs	Downregulated DEGs	Upregulated DEGs
A1 vs. A2	4265	1936	2329
A1 vs. A3	3987	2236	1751
A1 vs. A5	4005	2431	1574
A2 vs. A3	4253	2507	1746
A2 vs. A5	3837	2540	1297
A3 vs. A5	3629	2087	1542
A4 vs. A1	4456	1679	2777
A4 vs. A2	4095	1314	2781
A4 vs. A3	2881	1176	1705
A4 vs. A5	2152	1146	1006

**Table 3:** The hub genes detected in the purple and violet modules.

Module	Gene	Annotation	Connectivity
Purple	Smechr0303487	Homobox-leucine zipper protein GLABRA2	164.2872382
Purple	Smechr1201797	Flavonoid 3′5′-hydroxylase	163.1378627
Purple	Smechr0302054	Uncharacterized protein	163.0477793
Purple	Smechr0601814	GATA transcription factor 26	162.7337461
Purple	Smechr1002540	Anthocyanin 3-O-glucosyltransferase	159.0606892
Purple	Smechr0500713	Phenylalanine ammonia-lyase 2	157.7681631
Purple	Smechr1002420	WRKY transcription factor 44	157.7222576
Purple	Smechr1102209	Kirola	157.0941481
Purple	Smechr0901701	Anthocyanin-related transcription factor TT8	156.9502973
Purple	Smechr0302347	4-coumarate-CoA ligase 2	156.3548796
Violet	Smechr1000379	Chlorophyll a-b binding protein	103.30002
Violet	Smechr0902639	30S ribosomal protein S1	102.53807
Violet	Smechr0901976	Uncharacterized protein	101.83604
Violet	Smechr0502558	Protein TIC55, chloroplastic	101.50027
Violet	Smechr1100719	ABC transporter F family member 5	98.330714
Violet	Smechr0900664	Chlorophyll a-b binding protein	97.539107
Violet	Smechr1001174	Uncharacterized protein	94.912891
Violet	Smechr1200635	Chlorophyllide a oxygenase	90.176381

## Data Availability

The data presented in this study are available on request from the corresponding author.

## Supplementary Materials

The following supporting information can be downloaded at: https://www.mdpi.com/article/10.3390/ijms232113475/s1, Figure S1: Orthogonal partial least squares discriminant analysis discriminant analysis (OPLS-DA) model of comparison groups including A1 vs. A2, A1 vs. A3, A1 vs. A5, A2 vs. A3, A2 vs. A5, and A3 vs. A5.; Figure S2: Differential expressed genes involving in plant hormone transduction; Figure S3: Gene network of the hub genes in the violet module, which positively correlated with total chlorophyll content (*r^2^* = 0.94, *p* = 1 × 10^−7^); Figure S4: Sequence variation of *SmMYB113* among white, green, and purple fruit of eggplant. Table S1: DEGs identified in the pair-wise comparisons; Table S2: DEGs related to light-harvesting chlorophyll protein complex; Table S3: Primers used for quantitative real-time PCR (qRT-PCR).
