# Supplementary material for: Integrated Metabolome and Transcriptome Analysis Reveals a Regulatory Network of Fruit Peel Pigmentation in Eggplant (Solanum melongena L.)
Source: Int J Mol Sci. 2022 Nov 3;23(21):13475. doi: 10.3390/ijms232113475 (PMC9657510; doi:10.3390/ijms232113475)
Supplement: Supplementary file 1 [file ijms-23-13475-s001.zip › IJMS2004102 supplementary files proof.pdf]

Supplementary materials

Supplementary Table S1. DEGs identified in the pair-wise comparisons

Supplementary Table S2. DEGs related to light-harvesting chlorophyll protein complex

ID	annotation	A1_FPKM	A2_FPKM	A3_FPKM	A4_FPKM	A5_FPKM
Smechr0500697	Lhca1	423.39	582.08	268.39	26.85	25.88
Smechr0500699	Lhca1	33.26	38.92	14.29	2.62	2.68
Smechr0400151	Lhca2	206.07	274.72	140.28	19.67	11.11
Smechr1200202	Lhca2	0.58	14.68	0.34	0.00	0.02
Smechr0400296	Lhca3	153.72	237.91	121.20	15.87	9.53
Smechr0303053	Lhca4	286.63	350.87	145.82	18.38	10.73
Smechr0701037	Lhca5	17.17	42.56	21.35	10.71	7.75
Smechr0801141	Lhcb1	68.68	101.22	16.88	0.06	0.05
Smechr0801142	Lhcb1	2358.65	3117.48	1297.21	31.87	7.02
Smechr1000379	Lhcb3	209.30	166.36	88.26	9.19	3.01
Smechr0900664	Lhcb4	274.51	237.36	148.14	22.85	9.73
Smechr0602085	Lhcb5	109.34	158.79	115.57	10.11	5.11
Smechr0100613	Lhcb6	72.55	151.10	65.22	1.79	0.56
Smechr0100616	Lhcb6	90.47	101.71	56.47	4.19	2.59
Smechr0402609	Lhcb7	14.79	11.91	8.23	8.82	4.82

Supplementary Table S3. Primers used for quantitative real-time PCR (qRT-PCR)

Genes	Forward primer (5’ to 3’)	Reverse primer ((5’ to 3’)
Smechr0702290	TCATCCGACGAATGTTTTCA	GTCCCGAAGTGTTGTTGGAT
Smechr0802143	CCCAATTTACCACGGTGTTC	CAGCCATTTCAGGTGTAGCA
Smechr0802451	CGGCGAGTAAGCTAAAGGTG	ATAGCCGCGTGGTCATAAAC
Smechr0900664	GATTCGGATTCGGACAGAAA	ATCCGTAGTCACCGACAAGG
Smechr1001846	AAGCCAGAAGCAGATGTCGT	AGCTGCACCTCCAGCTACAT
Smechr1002423	GATGGGGCAGTTACTTGGAA	CTGGACCCAAAGTTCGTGAT
Smechr1200635	CCCTCTTGATCTTGGCTCAG	CTGCAGGTGGATCATTTCCT
Smechr1002213	AGTGGCATCTTGTTCCTGCT	CCGAAGCAAAGTCACCTCTC
Smechr1002420	GGCCAGATTGCTGAAATTGT	GGCATTGCCTAGAGTTGCTC
Smechr1002081	GGTGGACCACAAACAATGAA	GCTGAAATTTCTCCCCATGA
Smechr0400031	ACAGGAGGTGCAGGCTACAT	TTCATCTGCACCACTGAAGC
Smechr0401078	TGGCCTAAAAACCCTCCTTT	CAGCTGCCTCAATCATTTCA
Smechr0303487	CATTGTGGACGTTTCTGTGG	TGCATTGGATTGTTCTTGGA
Smechr0500713	GTTTTGCCCAAGGAGATTGA	AGCCCCTAATTCTTCCCTCA
Smechr0302347	TTCGAGATAGGTGCGCTTTT	GCTTGCGGGACTCTTCTATG
Smechr0601814	TCTTTTGCACCCCCAGATAG	CCACCCACTTGGACTTTGTT
GAPDH	CCGCTCCTAGCAAAGATGCC	ACCCTCCACAATGCCAAACC

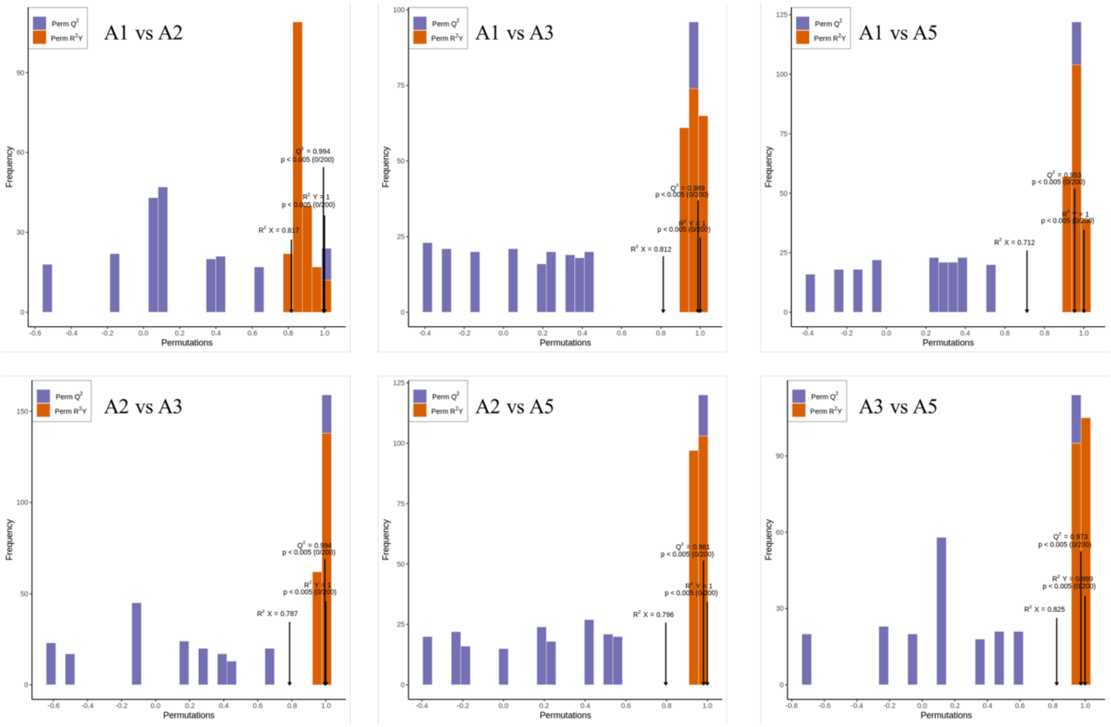

Supplementary Figure S1. Orthogonal partial least squares discriminant analysis (OPLS-DA) model of comparison groups including A1 vs. A2, A1 vs. A3, A1 vs. A5, A2 vs. A3, A2 vs. A5, and A3 vs. A5.

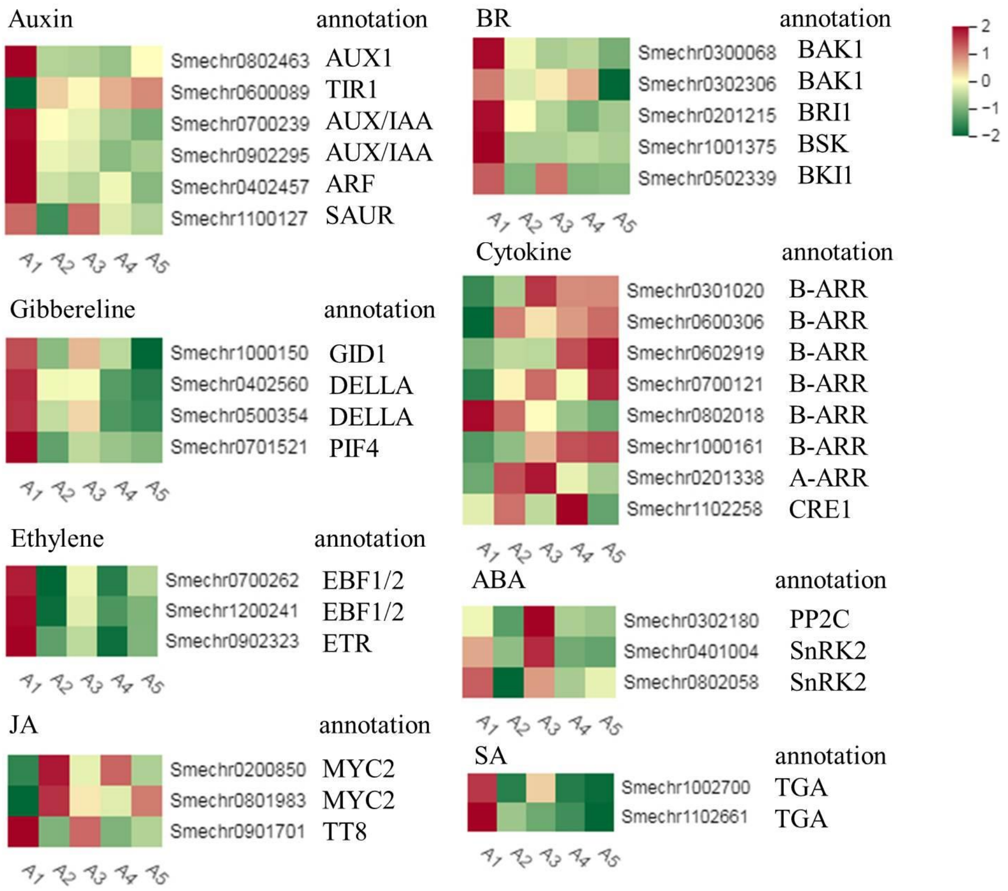

Supplementary Figure S2. Differentially expressed genes involved in plant hormone transduction.

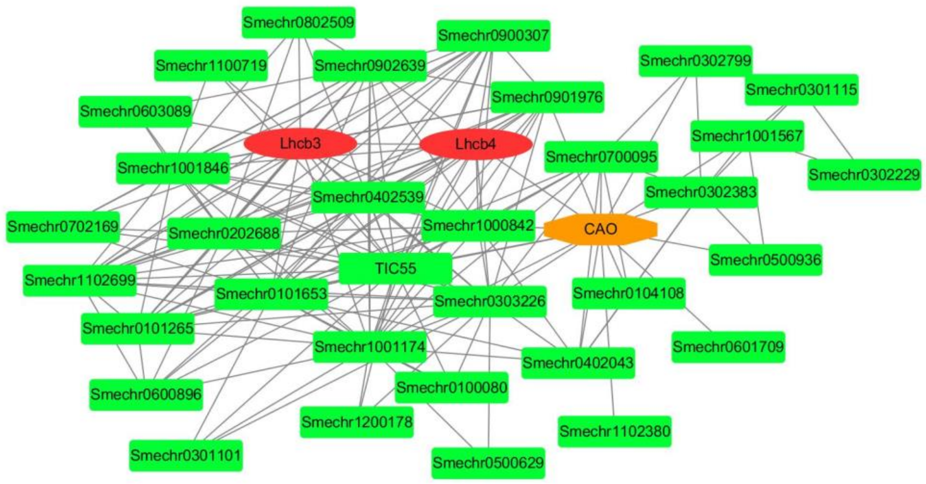

Supplementary Figure S3. Gene network of the hub genes in the violet module, which positively correlated with total chlorophyll content (r^2=0.94, p=1× 10^{-7}).

```
A1        MNNPPIICTSVRVRKGSWTEEEDLLLRKCMEKYGEGKWHLVPARAGLNRCRKSCRLRWLN
A2        MNNPPIICTSVRVRKGSWTEEEDLLLRKCMEKYGEGKWHLVPA..GLNRCRKSCRLRWLN
A3        MNNPPIICTSVRVRKGSWTEEEDLLLRKCMEKYGEGKWHLVPARAGLNRCRKSCRLRWLN
A4        MNNPPIICTSVRVRKGSWTEEEDLLLRKCMEKYGEGKWHLVPA..GLNRCRKSCRLRWLN
A5        MNNPPIICTSVRVRKGSWTEEEDLLLRKCMEKYGEGKWHLVPARAGLNRCRKSCRLRWLN
green1    MNNPPIICTSVRVRKGSWTEEEDLLLRKCMEKYGEGKWHLVPA..GLNRCRKSCRLRWLN
green2    MNNPPIICTSVRVRKGSWTEEEDLLLRKCMEKYGEGKWHLVPA..GLNRCRKSCRLRWLN
green3    MNNPPIICTSVRVRKGSWTEEEDLLLRKCMEKYGEGKWHLVPA..GLNRCRKSCRLRWLN
purple1   MNNPPIICTSVRVRKGSWTEEEDLLLRKCMEKYGEGKWHLVPARAGLNRCRKSCRLRWLN
purple2   MNNPPIICTSVRVRKGSWTEEEDLLLRKCMEKYGEGKWHLVPARAGLNRCRKSCRLRWLN
purple3   MNNPPIICTSVRVRKGSWTEEEDLLLRKCMEKYGEGKWHLVPARAGLNRCRKSCRLRWLN
white1    MNNPPIICTSVRVRKGSWTEEEDLLLRKCMEKYGEGKWHLVPA..GLNRCRKSCRLRWLN
white2    MNNPPIICTSVRVRKGSWTEEEDLLLRKCMEKYGEGKWHLVPA..GLNRCRKSCRLRWLN
white3    MNNPPIICTSVRVRKGSWTEEEDLLLRKCMEKYGEGKWHLVPA..GLNRCRKSCRLRWLN
Consensus mnnppiictsvrvrkgswteeedlllrkcmekygegkwhlvpa  glnrcrkscrlrwln
```

Supplementary Figure S4. Sequence variation of *SmMYB113* among white, green and purple fruit of eggplant.
